# Machine Learning for AI Breeding in Plants

**DOI:** 10.1093/gpbjnl/qzae051

**Published:** 2024-07-02

**Authors:** Qian Cheng, Xiangfeng Wang

**Affiliations:** State Key Laboratory of Maize Bio-breeding, National Maize Improvement Center, Frontiers Science Center for Molecular Design Breeding, China Agricultural University, Beijing 100094, China; State Key Laboratory of Maize Bio-breeding, National Maize Improvement Center, Frontiers Science Center for Molecular Design Breeding, China Agricultural University, Beijing 100094, China

What makes artificial intelligence (AI) smart is machine learning (ML), which is defined as “a field of study that gives computers the ability to learn without being explicitly programmed” by ML pioneer Arthur Samuel in 1959. ML deduces data patterns without relying on prior assumptions as statistics does, greatly reducing the human effort required to comprehend the data. ML comprises a large family of algorithms, many of which support big data analytics [1]. With the rapid advances in multi-omics technologies, plant breeding has entered the “genome, germplasm, genes, genomic breeding, and gene editing (5G)” generation [2], in which biological knowledge and omics data are integrated to expedite trait improvement. ML holds great promise for 5G breeding, with many reports of ML applications for omics-driven gene discovery, genotype-to-phenotype (G2P) prediction, genomic selection (GS), and plant phenomics. However, there remains a gap between basic research and breeding practices in plants [3]. Given that multi-omics, genotypic, phenomic, and environmental datasets have become highly dimensional and heterogeneous, novel ML algorithms are expected. Hereby, we propose ways to overcome major challenges in the application of cutting-edge ML models to plant research, with the ultimate goal of making plant breeding smart and easy.

## Population-scale multi-omics analysis for gene discovery

Discovery of agronomically useful genes is the premise for exploiting natural variations for marker-assisted selection (MAS) or creating artificial mutations via genome editing. Genome-wide association studies (GWAS) of common agronomic traits have reached a bottleneck, as their power to dissect complex, polygenic traits is quite limited. Multi-omics analysis focusing on a reference germplasm panel under different spatiotemporal conditions could greatly enhance the mapping resolution of causal genes and mutations when cellular biomolecules (*e.g.*, RNA transcripts, proteins, metabolites) are treated as molecular traits (mTraits). Additionally, phenomics has become another main component in multi-omics, in which phenomic data are mostly generated by high-throughput imaging equipment using computer vision technologies. Since phenomic features may reflect certain physiological activities inside plant cells, this type of feature can be regarded as imaging traits (iTraits).

### Coping with the “curse of dimensionality”

Population-scale multi-omics datasets tend to be highly dimensional, noisy, and heterogeneous. This issue is addressed using a type of unsupervised learning known as dimensionality reduction (DR) to prevent the “curse of dimensionality”. The Multi-Omics Data Association Studies (MODAS) toolbox applies multiple DR algorithms to genotypes and mTraits in plants [4]. To perform DR on genotypes, MODAS combines the Jaccard similarity coefficient, density-based spatial clustering of applications with noise (DBSCAN), and principal component analysis (PCA) algorithms to generate a “pseudo-genotype index” file. This highly simplified variation atlas uses tens of thousands of genomic blocks to represent millions of single-nucleotide polymorphisms (SNPs) in the genome, improving analytical efficiency for mapping mTraits.

The dimensionality of mTraits must also be reduced, as omics data are highly redundant due to technical issues and the characteristics of biological pathways. For example, a metabolite is produced by a cascade of enzymatic reactions involving many genes and pathways, and crosstalk between pathways is common. Therefore, given their highly correlated pattern, both final products and intermediate compounds could be repeatedly mapped to the same region. The non-negative matrix factorization (NMF) algorithm removes redundancy by decomposing the matrix of metabolites_(__*n*__)_ × samples_(__*m*__)_ into one meta-metabolite dimension and one meta-sample dimension. The weights of a meta-metabolite across samples represent the overall abundance of a set of clustered compounds, and the weights of meta-samples reflect subgroups of samples divided based on the haplotypes of the mapped region. The genomic blocks that contribute to the corresponding biosynthetic pathway are mapped via GWAS between the meta-metabolites and the pseudo-genotype index. SNPs within the block are then used to identify causal genes and mutations. This strategy greatly reduces computing time and saves resources while providing clean, easy-to-interpret results.

### Automated feature engineering

Another common issue is that feature sets, such as SNPs, mTraits, or iTraits, are far larger than sample sets. This increases the risk of overfitting, as the model may learn incorrect features from the data. Thus, feature engineering, including feature selection or feature extraction, must be performed before training a model. Feature selection tends to select a small subset from the total features without changing the original feature values. This can be achieved by manual selection based on prior knowledge or automated selection by learning the importance of features when training a model. By contrast, feature extraction creates a small set of new features by summarizing the characteristics of the original features. NMF is a form of feature extraction, as meta-metabolites are new features derived from a much larger set of metabolites. Feature engineering can be embedded in many ML paradigms, such as deep learning (DL) and ensemble learning (EL). The DL convolutional neural network algorithm performs feature extraction when transferring information between network layers. Light Gradient Boosting Machine (LightGBM) performs feature selection by computing a score of information gain (IG) to select features of high importance.

Although automated hyperparameter tuning using grid searches is widely implemented in plants, automated feature engineering has largely been neglected. In a recent study, SNP features with high IG scores selected by LightGBM were consistent with the peak SNPs identified from GWAS, indicating the ability of the algorithm to recognize trait-associated variations [5]. It suggests that automated feature selection can also be used to discover agronomically important genes and to facilitate panel design of compiling effective molecular markers associated with traits of interest for MAS. In addition to methods embedded in ML algorithms, many independent tools specifically designed for feature engineering could also be utilized in plants, such as the deep feature synthesis method in the Python “Featuretools” library.

### Manifold learning for data visualization

Manifold learning uses non-linear DR algorithms to visualize datasets with ultrahigh dimensionality, which helps maintain the geometric properties of high-dimensional data, even when mapped to a low-dimensional space. This technique is especially useful for visualizing single-cell RNA sequencing (scRNA-seq) data. Multiple algorithms have been utilized to investigate the structures of heterogeneous cell populations based on scRNA-seq data, including *t*-distributed stochastic neighbor embedding (*t*-SNE), Uniform Manifold Approximation and Projection (UMAP), and Potential of Heat-diffusion for Affinity-based Trajectory Embedding (PHATE). Another strategy utilizes deep neural networks (DNNs) to extract information from internal nodes at different network layers to simultaneously achieve batch correction, clustering, denoising, and data visualization under a unified model. DL using this strategy is no longer regarded as a “black box”, as the geometric properties may reflect the biological features extracted by the hidden layers of DNNs. Sparse Autoencoder for Unsupervised Clustering, Imputation, and Embedding (SAUCIE) performs DR and visualization of scRNA-seq data simultaneously. Other omics data types have also been generated at single-cell resolution. Aligning and integrating multiple levels of omics data for the same cell populations has become a new challenge.

### Fine-mapping of causative variants

In essence, gene discovery is to identify allelic genomic variations that are beneficial to a designated trait. Thus, fine-mapping of causative variants, including SNPs, insertions and deletions (InDels), presence and absence variations (PAVs), and a variety of structural variations (SVs) causing direct functional change, is important for precision-designed breeding. This is especially true for improving qualitative traits determined by single gene with major effect. However, causative variations involving coding SNPs or short InDels that alternate protein functions only account for a very small fraction of trait-related variations. Mapping of regulatory variants attributable to SVs and PAVs is much difficult, as it requires high-quality pan-genome sequences derived from *de novo* assembly of representative core germplasm lines. To achieve this goal, multiple steps assisted by different types of omics data are required. It first starts with rough-mapping of a genomic interval, usually ranging in megabases, by GWAS analysis of the target trait; then, integrative analysis of various datasets generated from transcriptome-wide association study (TWAS), metabolome-wide association study (MWAS), and other type of techniques profiling *cis*-regulatory elements by chromatin immunoprecipitation sequencing(ChIP-seq) or self-transcribing active regulatory region sequencing (STARR-seq) has to be done to further narrow down the list of candidate genes or genomic regions; third, genotypes of SNPs in the candidate genes and regions are mapped to pan-genome assembly to determine the haplotypic map (HapMap) associated with each of the SVs or PAVs; at last, statistical testing is performed to examine whether the PAV-associated HapMap is significantly consistent with phenotypic variations. However, it’s worthy of noting that these so-called causative variants identified from multi-omics analysis are only candidate genes or variations. Whether they are directly involved in functional variations contributing to trait change still requires strict experimental validation, before this functional marker can be finally utilized in molecular design breeding. Because fine-mapping of causative variants involves multiple forms of population-scale omics data which are recently defined as panomics by Weckwerth et al., development of ML methods solving integrative analysis of panomics has been highly expected [6].

## Knowledge-driven molecular design breeding

Knowledge from plant research should ultimately facilitate applied plant breeding. With an explicit understanding of the biological mechanisms underlying a trait, the causal gene can be precisely utilized for trait improvement. Yet, translating biological knowledge into breeding remains challenging. For example, germplasm panels used for GWAS usually consist of wild relatives, landraces, obsolete cultivars, and modern cultivars to ensure genotypic and phenotypic diversity. However, most mutations mapped in germplasm are no longer present in modern cultivars, as deleterious alleles have been removed and beneficial alleles fixed by artificial selection. Hence, relatively few genes are utilized in modern breeding, and mutations in these genes conveying desirable traits usually vary from population to population. A foreground mutation only functions properly under a specific genetic background; thus, even if a mutation discovered from germplasm is potentially valuable, it may not be directly utilizable in modern breeding systems. Similarly, when creating artificial mutations, the new mutation must adapt to the existing gene regulatory networks. Therefore, the bottleneck is not the genome editing or transgenic techniques but rather the need to identify genes and recipient materials that can be modified without affecting non-target traits.

### Breeding is all about “timing” and “balancing”

Trait improvement is essentially a process of fine-tuning a gene regulation network. Crossing generates new patterns of gene regulation by recombining deleterious and beneficial alleles. This process offers the chance to select the optimal network where genes involved in a regulatory pathway meet the breeding goal of trait improvement. Thus, even a small phenotypic change might involve a reshaped gene regulatory network influencing complex interactions among genes and pathways. It is also important to clarify the definition of deleterious and beneficial alleles. That is, no allele is absolutely deleterious or beneficial: alleles are defined based on their final effects on yield. However, deleterious and beneficial statuses are potentially interconvertible depending on the developmental stage and/or environment. For example, a beneficial allele for vegetative growth is beneficial for biomass accumulation but may be deleterious to yield-related traits by negatively affecting reproductive development [7]. Thus, breeding cannot be simply understood as a way of removing deleterious alleles or pyramiding beneficial alleles; instead, the effects of two sets of counteracting alleles must be balanced.

How can our knowledge of genes and mechanisms be efficiently translated into applied breeding? ML is suited for this mission due to its capacity to integrate knowledge and data. To illustrate this, consider ML-facilitated molecular design to breed maize cultivars suitable for mechanical harvesting. This requires considering multiple traits for improvement, including plant compactness, kernel dehydration rate, times of flowering and maturity, stalk stiffness and strength, and corn husk morphology. The greatest difficulty is dealing with the pleiotropic effects of genes: changing one trait may affect other traits. Target-oriented prioritization (TOP), a recently developed integrative multi-trait ML algorithm, mathematically learns the synergistic or competitive relationships among multiple traits to make a cohesive decision for selecting superior candidates [8]. As long as sufficient genotypic and phenotypic data are acquired, ML models can establish the correlations between genes and traits based on knowledge graph. The target genes for a designated breeding population can be assembled as a panel for ML algorithms to learn the optimal pattern of allelic combinations. The model then aids the selection of materials with the desired haplotypes to simultaneously improve multiple traits.

### Panel design with EL

Genotyping by targeted sequencing (GBTS), which captures SNP-containing regions for gene panel sequencing, is widely used for genetic diagnostics in precision medicine. A typical GBTS panel contains thousands to tens of thousands of SNPs covering dozens to hundreds of genes, allowing hundreds of samples to be multiplexed for genotyping. However, the cost per sample of genotyping is still relatively high for plant breeding because of the need to process tens of thousands of samples. Nonetheless, GBTS is a good method for accumulating training data for ML until the population is large enough to cover all possible allelic combinations of target genes. As long as the most stable SNPs are identified, a new low-cost panel containing dozens of SNPs could be designed.

Ultrahigh-throughput, scalable platforms based on kompetitive allele-specific PCR (KASP), such as Nexar Array Tape systems, could then be utilized. These platforms can multiplex tens of thousands of samples per run, but the markers must be highly universal and effective. One can then take advantage of feature selection embedded in EL to select markers. EL is a family of ML algorithms, including random forest, gradient boosting decision tree (GBDT), extreme gradient boosting (XGBoost), categorical boosting (CatBoost), and light gradient boosting machine (LightGBM), which assemble outcomes from multiple weak learners to enhance predictability. LightGBM generates leaf-wise trees and identifies the “best leaves”, which in this case are SNPs with high utility for classify traits. This ability is represented by an IG score, which resembles the effect of the SNP inferred from GWAS [5]. Therefore, LightGBM is an ideal tool for compiling highly condensed panels of SNPs via automated feature selection while maintaining maximum predictability.

### Pathway design via causal learning

While a marker panel covers SNPs associated with relevant traits identified from GWAS analysis, a pathway panel may contain variations associated with genes forming a regulatory network or located in a metabolic biosynthesis pathway identified from multi-omics analysis. Therefore, designing a pathway panel requires the inference of the “cause” and “effect” relationship between two genes, such as a transcription factor and a target gene. Compared to the marker panel that is usually used for improving regular agronomic traits covering thousands of SNP markers, a pathway panel may contain much less markers associated with genes for improving specific characteristics of plants, such as anti-stress feature or enhancing the content of certain metabolite compounds. The inferred causality can be used as a rule to design a trait panel by clustering functionally related genes. Mendelian randomization (MR) was recently used to infer the causal relationships between mutations, genes, biomolecules, and traits in plants based on summarized results from population-scale multi-omics analysis [4]. However, the assumption underlying MR is based on human population genetics. Whether this tool is applicable to all plant species requires validation, as domesticated plants result from artificial selection rather than natural selection. It is therefore necessary to seek novel methods independent of genetic assumptions. In fact, ML and causal inference are two independent fields with different methodological systems: ML predicts outcomes based on data correlations without explaining causality, whereas causal inference determines the roles of the “cause” and “effect” of variables. Data scientists are trying to combine these two systems. The new field of “causal learning” confers the ability of ML models to explain underlying reasons, thereby making AI more closely resemble real-world decision-making. For example, causal representation learning was designed to discover high-level causal variables based on low-level observations. Causal tree learning, a modified version of the classification and regression tree (CART) model, estimates causal relationships during the process of tree splitting. These methods could be used to reconstruct biological networks from multi-omics data, in which the inferred causalities represent the directional edges among nodes.

## Data-driven genomic design breeding

Data acquired from industrial breeding programs can include genotypic, phenotypic, environmental, climate, and any type of field data. Unlike knowledge-driven design, data-driven design does not require knowledge of the specific genes and mechanisms underlying a trait. Instead, it uses statistical or ML models to infer correlations among data, as exemplified by GS [9]. However, genotyping cost is still the main factor hindering the wide application of GS in plant breeding industry. A promising substitution of GBTS is low-coverage genome-wide sequencing (lcGWS) or ultra-low-coverage genome-wide sequencing (ulcGWS) which randomly sequences genomic DNA at an expected coverage of 1.5× or 0.5×, respectively. Genotyping cost by lcGWS is much lower than GBTS, since it skips the step of capturing targeted DNA fragments. Nevertheless, because DNA fragments are randomly sequenced by lcGWS, SNPs may not be consistently covered by all the genotyped samples. One possible solution is to first construct a reference HapMap composed of all elite inbred lines, which includes usually 50 to 100 lines frequently used as founder lines to generate doubled haploid (DH) lines in a breeding project. However, the reference HapMap has to be constructed with high-coverage genome-wide sequencing (hcGWS; *i.e.*, 30×), so that it can be used to perform imputation on genotypic data of DH lines that are generated by the founder lines included in the HapMap. By this means, a relatively consistent SNP panel can be inferred to perform GS prediction. It’s worthy of attention that, because genotypes of SNPs inferred by imputation may include a fraction of inestimable mistakes, the DH lines are better descendants or close relatives of the founder lines included in the HapMap, and strict SNP filtration must be done before imputation is performed in order to minimize the fraction of wrong genotype information.

With the help of decision-making models, inputs from human experience are largely minimized in a breeding pipeline. The main purpose is to reduce costs, and precision is not the top priority. Thus, the balance between cost and precision must be considered in actual breeding practices. As the cost of genotyping and phenotyping accounts for the main proportion of total expense in a breeding project, a GS project usually uses 20%–25% of the entire population to obtain both genotypic and phenotypic data to construct training dataset. Under this ratio of training and testing samples, yield prediction accuracy may achieve from 0.5 to 0.6 according to the evaluation of Pearson correlation coefficient, but the total cost may be approximately reduced 30% to 40%. For example, a pilot maize breeding project used ∼ 9000 hybrids to train a GS model and predicted the trait performance of ∼ 34,000 untested hybrids, providing an in-depth understanding of the genetic mechanisms of heterosis and cross combinations for subsequent breeding cycles [6]. Another common issue in GS is population stratification when multiple panels of distantly related germplasm are involved in crossing. The proper partitioning of the training and prediction samples must be carefully considered to prevent serious overfitting.

More and more studies have illustrated the feasibility of integrating multi-omics data to further improve prediction precision based on DL or DNN to facilitate GS or genomic prediction (GP), such as the tools of DeepGS and DNNGP [10,11]. However, direct use of multi-omics data in training a GS model is risky, as it may cause inestimable overfitting due to the extremely high complexity of feature sets. Therefore, aforementioned feature engineering on mTraits or iTraits must be utilized to reduce data dimensionality prior to model training. Then, the dimensional vectors are regarded as features to be incorporated with genotypes of SNPs to train GS models. Additionally, generation of multi-omics data is costly and it’s impossible to generate RNA sequencing (RNA-seq) or metabolome profiling for each individual samples in each breeding cycle. We should only utilize the biological information derived from a set of multi-omics data, which is essentially the innate correlation of different omics datasets. Therefore, transferring learning with interpretable DL framework is promising to transfer the network layers derived from multi-omics data to be integrated with genotypes of SNP features. By this means, issues of sequencing cost and data complexity can be both properly solved.

A commercial breeding pipeline can be partitioned into multiple stages, and each stage may generate data for building decision-making models. In theory, any problem solved by statistical models can also be solved by ML. However, thus far, only GS has been implemented using ML methods, and most other studies have been based on statistics. GS is widely employed for maize breeding due to the use of single-cross breeding in the modern maize industry: in this situation, genotyping parental inbred lines makes it possible to infer the F_1_ genotypes, greatly reducing genotyping costs. However, attention should be paid to the utility of GS for the breeding goals. GS is suitable for interrogating the general combining ability or heterotic performance between two parental pools using genome-wide genetic background, since heterosis is determine by genomic kinship rather than a few markers. Thus, the ultimate goal of GS is to accelerate the progress of genetic gain using *in silico* prediction to reduce field costs. Nevertheless, if the goal is to fine-tune a specific trait, such as the ability of stress tolerance, GS is unsuitable, while the ideal solution is molecular design breeding using a small set of trait-associated markers (also called genetic foreground) after the causal genes mapped.

Because GS may not solve all problems encountered in breeding, complementary models have been developed. For example, genome optimization via virtual simulation (GOVS) utilizes least-squares means to infer genomic fragments with beneficial effects on grain yield and simulates an assembly of all beneficial fragments as an optimized genome [12]. The simulated genome facilitates the selection of superior lines based on the number of beneficial fragments rather than the predicted phenotype. GOVS also helps identify lines with complementary sets of beneficial fragments. These complementary lines can be crossed, and doubled haploid technology can be used to precisely pyramid beneficial fragments.

Modeling the phenotypic plasticity of plants in response to the environment is another important way to facilitate decision-making during breeding. Phenotypic plasticity results from genotype–environment interactions (G×E) [13]. The G×E model helps identify the optimal ecological range for achieving the highest yield productivity and estimates yield stability across different ecological zones. If more complicated climate factors are considered, the model also helps estimate the influence of climate change on yield performance and grain quality and identifies the optimal genotypes adapted to climate change. However, most methods for modeling G×E are based on linear regression algorithms to infer correlations between yield performance and a few environmental factors. Statistical models have become unsuitable for modeling increasingly complicated genotypic, phenotypic, environmental, and climate datasets, prompting the need for ML methods. Another critical issue for modeling phenotypic plasticity is heterogeneous plasticity between inbred lines and hybrids, which strongly influences model precision and must be considered when using ML methods to predict environment-specific traits from inbred to hybrid lines.

Although in theory, all problems solved by statistics can be all solved by ML, ML is not always the best choice. If the problem is a “white box”, statistics should be used, especially when the number of explicitly labeled samples is insufficient to cover all patterns that can be learned by an ML model. If the training dataset is smaller than the testing dataset, an ML model will usually have lower prediction precision than a statistical model. The scarcity of labeled samples is a common issue in breeding, not only because phenotyping is costly and labor intensive, but also because certain traits are difficult to explicitly define and accurately measure, such as biotic and abiotic stress-related traits. Semi-supervised learning is a promising method for coping with this issue, including positive-unlabeled learning, generative adversarial network, contrastive learning, and transfer learning, but requires caution in its application [14]. If the data distribution is not uniform, inestimable overfitting may occur, as the bias will be amplified by predicted labels. Another option is multi-modal learning, which integrates complementary information in multiple modalities to discover a latent representation of the data. Joint DR (jDR) was effectively used to integrate multi-source transcriptome, copy number variation (CNV), microRNA, and methylome data from the same sample for human cancer prediction and classification. With the rapid generation of omics data from plant germplasms, perhaps this multi-modal learning algorithm could be used to address the problem of a limited sample size for model training.

## Building an ecosystem for AI breeding in plants

**Figure 1:**
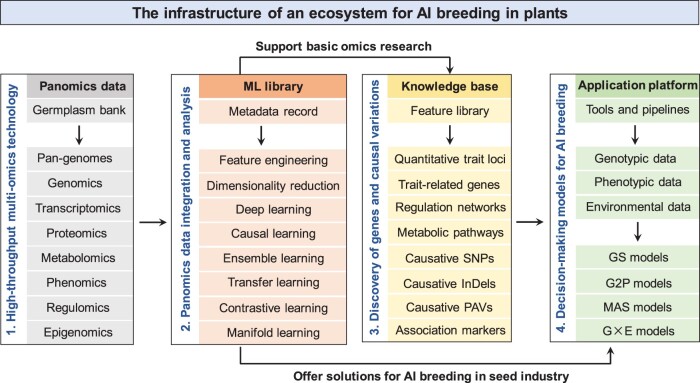
The infrastructure of an ecosystem for AI breeding in plants The proposed ecosystem is composed of four major components. The first component is a data center that contains all types of multi-omics data generated from a representative germplasm bank. The second component is a library of cutting-edge ML algorithms that can be used to either support basic omics research in plants or offer solutions for building decision-making models in the seed industry. The third component is a knowledge base that contains trait-related genes and causal variations derived from multi-omics data association analysis. The last component is an application platform that contains a spectrum of bioinformatics tools and statistics-/ML-based decision-making models for AI breeding. AI, artificial intelligence; ML, machine learning; SNP, single nucleotide polymorphism; InDel, insertion and deletion; PAV, presence and absence variation; GS, genomic selection; G2P, genotype-to-phenotype; MAS, marker-assisted selection; G×E, genotype–environment interaction.

A common consensus is that high-quality datasets and labels are more important than ML models themselves. This rule also applies to breeding. A recent study evaluating 12 GS models by predicting 18 traits in six plant species showed that no single method performed best across all traits and species [15]. Hyperparameter tuning is essential for achieving the best performance using ML. This study revealed the complications of applying ML to plant breeding, perhaps due to the complex composition of genetic materials and the influence of the environment on phenotypes. Thus, precision is not the only goal when applying ML to breeding: the robustness, extendibility, and efficiency of a model must also be considered. An ML ecosystem specifically designed for AI breeding in plants is highly anticipated by the seed industry. This ecosystem must contain three major components: data, model, and application platforms (**Figure 1**). The data platform should consist of unified pipelines for automated collection, processing, analysis, and storage of genotypic and phenotypic data, facilitated by cloud-based computing. The model platform will include GS, G2P, G×E, and other decision-making models developed using ML and statistical methods, with automated modules used for model selection, feature engineering, and hyperparameter tuning. The application platform will consist of tools implemented from the predictive models, equipped with a user-friendly interface to offer services and report results to end users. Such an ML ecosystem will make plant breeding smarter and easier in this era of AI.

## CRediT author statement

**Qian Cheng:** Writing – review & editing. **Xiangfeng Wang:** Conceptualization, Writing – original draft, Writing – review & editing, Supervision. Both authors have read and approved the final manuscript.

## Competing interests

Both authors have declared no competing interests.
